# Pregnancy Monitoring

**DOI:** 10.1155/2014/507613

**Published:** 2014-03-04

**Authors:** M. Mischi, B. Karlsson, M. G. Signorini, M. Ungureanu, C. Marque

**Affiliations:** ^1^Eindhoven University of Technology, Electrical Engineering Department, Signal Processing Systems, Biomedical Diagnostics Research laboratory, Den Dolech 2, 5612 AZ Eindhoven, The Netherlands; ^2^Reykjavik University, School of Science and Engineering, Institute of Biomedical and Neural Engineering, Menntavegur 1, 101 Reykjavik, Iceland; ^3^Politecnico di Milano, Dipartimento di Elettronica, Informazione e Bioingegneria DEIB, Piazza Leonardo da Vinci 32, 20133 Milano, Italy; ^4^Politehnica University of Bucharest, Applied Electronics and Information Engineering Department, Iuliu Maniu 1-3, 060042 Bucharest, Romania; ^5^Université de Technologie de Compiègne, UMR CNRS 7338, Biomécanique et Bioingénierie, rue Roger Couttolenc, CS60319, 60203 Compiègne Cedex, France

Pregnancy is a happy but also very risky time, especially for the fetus. Fetal distress and preterm birth make this the most dangerous period in a person's life. Indeed the risk of death for the fetus during the last third of pregnancy is similar to the accumulated risk of death in traffic during the rest of a person's life. Early assessment of these risks enables timely and effective intervention, which is essential to minimize perinatal mortality and long term morbidity. With this aim, this issue presents modeling and signal processing methods for reliable pregnancy monitoring, possibly overcoming current diagnostic limitations due to motion artifacts, low signal-to-noise ratio (SNR), and complex signal and image interpretation. In particular, the authors have focused on three main technologies, namely, ultrasound (imaging and Doppler), fetal electrocardiography (fECG), and uterine electromyography, referred to as electrohysterography (EHG). In fact, improved pregnancy monitoring for accurate assessment of both fetal condition and uterine activity requires advances in all of these technologies. In addition, specific methods for reduction of artifacts and interference in biopotential recordings are also presented and discussed aiming at producing data of better quality, which are suitable for reliable analysis.

In the area of ultrasound Doppler analysis of the heart rate, the paper “*New estimators and guidelines for better use of fetal heart rate estimators with Doppler ultrasound devices*” investigates a number of heart rate estimators, with the aim of achieving an accuracy of 0.25 beats per minute, required for reliable analysis of heart rate parameters. Based on the comparison of several estimators with simulations and 580 min recordings, only those estimators based on directional envelopes (using quadrature and in-phase components) and autocorrelation delay estimation could achieve the target accuracy. Moreover, combination of directional signals provided 14% increase in sensitivity, suggesting the implementation of a multitransducer approach for parallel processing as a valid strategy to pursue. Moving to ultrasound imaging, the paper “*Automatic evaluation of progression angle and fetal head station through intrapartum echographic monitoring*” provides a new automatic and reliable option for assessment of labor progression, without the need for digital inspection of the cervix. Progression angle and fetal head station could successfully be assessed in 10 women during labor with accuracy superior to standard methods. Earlier identification of abnormal labor patterns could therefore be achieved, supporting clinical decision.

Analysis of the fECG is complicated by the mixture of signals originating from different sources that contribute to the recorded signals, resulting in low-SNR fECG. The paper “*Probabilistic source separation for robust fetal electrocardiography*” proposes a probabilistic framework where blind source separation incorporates and exploits prior knowledge based on physiological modeling. The superiority of the approach as compared to standard blind separation methods is proven by simulation and multichannel fECG recordings. The fetal heart rate variability (FHRV) is among the most relevant features that can be extracted from the fECG. The paper “*Monitoring fetal heart rate during pregnancy: contributions from advanced signal processing and wearable technology*” proposes new diagnostic and classification indices based on advanced signal processing. Results on normal fetuses and intrauterine growth-restricted fetuses show that the estimation of different indices from FHRV signals, both linear and nonlinear, provides valuable indications to describe the pathophysiological mechanisms influencing the fetal heart rate. This paper also provides a perspective on wearable technology for fECG monitoring, focusing on the “Telefetalcare” system, using textile electrodes embedded in everyday garments. Still in relation to FHRV analysis for detection of fetal distress, nonlinear analysis tools are also proposed by the paper “*Coarse-grained multifractality analysis based on structure function measurements to discriminate healthy from distressed foetuses,*” where coarse-grained multifractal analysis of the fetal heart rate, using the Hurst exponent together with singularity and Holder spectra, shows promising results to discriminate healthy from distressed fetuses on 100 recordings.

EHG analysis is very promising and yet controversial. While evidence is being provided on the value of EHG analysis for detection of preterm labor, the complex mechanisms underlying the uterine activity are not fully understood yet. As a result, different groups are proposing different EHG parameters to characterize the uterine contractions. In the paper “*Comparison of different EHG feature selection methods for the detection of preterm labor,*” the best classification results (48 women) are obtained by nonlinear approaches, for either feature selection or classification, making also use of nonlinear features such as variance entropy. Different conclusions are somehow drawn in the paper “*Assessment of parturition with cervical light-induced fluorescence and uterine electromyography,*” where linear EHG features, such as propagation velocity and peak frequency, permitted prediction of the delivery time in a study with 88 patients. This paper also shows the value of light-induced fluorescence (LIF) of cervical collagen for monitoring labor progression, reflected in changes in the cervix compliance. Although with different velocity amplitudes, this result is in line with the paper “*Automated conduction velocity analysis in the electrohysterogram for prediction of imminent delivery: a preliminary study,*” where the amplitude of the EHG conduction velocity was found to be increased prior to (preterm) delivery based on the analysis of 22 patients. Moreover, the authors were able to automatically extract this feature from the EHG. In general, the controversial conclusions among the authors on the best EHG analysis may also be ascribed to the different geometry and configuration of the employed electrode grid, possibly sensitive to different EHG features evidenced at different spatial scales.

Dealing more specifically with artifacts and interference in biopotential recordings, accurate motion artifact detection is proposed in the paper “*Automatic identification of motion artifacts in EHG recording for robust analysis of uterine contractions,*” where detection is obtained with a classifier based on a total of 11 spectral, temporal, and nonlinear features, although the results suggest possible reduction to 7 features. The results were obtained with a Laplacian electrode configuration in 12 women in their first stage of labor. Another relevant noise source in the recording may be represented by the powerline. The paper “*Fetal ECG extraction from abdominal signals: a review on suppression of fundamental power line interference component and its harmonics*” presents a state-of-the-art review comparing the performance of digital notch filters, adaptive filters, Hilbert Huang transform, Wavelet transform, blind source separation, and neural networks with the Hilbert Huang transform method showing the best results for the implemented scenarios.

*M.  Mischi* *M.  Mischi* *B.  Karlsson* *B.  Karlsson* *M.  G.  Signorini* *M.  G.  Signorini* *M.  Ungureanu* *M.  Ungureanu* *C.  Marque* *C.  Marque*

